# Organ‐Specific Histopathological Effects of Prenatal Alcohol Exposure: A Narrative Review

**DOI:** 10.1002/cga.70047

**Published:** 2026-03-06

**Authors:** Rana Nur Gursu, Dilan Cetinavci, Hulya Elbe

**Affiliations:** ^1^ Department of Histology and Embryology, Faculty of Medicine Mugla Sitki Kocman University Mugla Türkiye; ^2^ Department of Histology and Embryology Mugla Training and Research Hospital Mugla Türkiye

**Keywords:** congenital anomalies, fetal alcohol spectrum disorders, fetal alcohol syndrome, neurodevelopmental disorders, oxidative stress, prenatal alcohol exposure, teratogenicity

## Abstract

The role of alcohol‐induced epigenetic modifications that predispose the fetus to metabolic dysregulation, increased susceptibility to future substance use, and long‐term behavioral and cognitive impairments has received increasing attention. Fetal alcohol syndrome (FAS) and fetal alcohol spectrum disorders (FASDs) remain underdiagnosed worldwide. As evidence continues to demonstrate that no safe level of alcohol consumption exists during pregnancy, strengthening public awareness, maternal education, and prenatal care services has become increasingly imperative. Prenatal alcohol exposure (PAE) induces distinct and reproducible histopathological alterations in multiple developing organs, including the brain, heart, liver, kidneys, lungs, eyes, limbs, and placenta. These alterations involve cellular degeneration, tissue disorganization, inflammatory infiltration, vascular abnormalities, and impaired organ architecture, arising from oxidative stress, inflammation, apoptosis, and disrupted developmental signaling during critical periods of fetal development. This narrative review provides a comprehensive synthesis of the organ‐specific histopathological consequences of PAE and integrates the underlying cellular and molecular mechanisms contributing to these alterations. Future research should further elucidate the molecular basis of ethanol teratogenicity and explore potential therapeutic strategies, such as antioxidant supplementation, nutritional modulation, and neuroprotective agents. Nevertheless, complete maternal abstinence from alcohol remains the most effective preventive strategy. By integrating histopathological findings across multiple fetal organ systems within a unified developmental framework, this review offers a distinct contribution beyond existing literature focused primarily on epidemiology or diagnosis.

**Abbreviations**
ADH: Alcohol Dehydrogenase
ADNF‐12: Activity‐Dependent Neurotrophic Factor‐12
ALDH2: Aldehyde Dehydrogenase 2
AMPA: α‐amino‐3‐hydroxy‐5‐methyl‐4‐isoxazolepropionic acid
ARBD: Alcohol‐Related Birth Defects
ARND: Alcohol‐Related Neurodevelopmental Disorder
BDNF: Brain‐Derived Neurotrophic Factor
BRIEF: Behavior Rating Inventory of Executive Function
CDC: Centers for Disease Control and Prevention
CNCC: Cranial Neural Crest Cells
CNS: Central Nervous System
CYP2E1: Cytochrome p450 2E1
DEAB: 4‐(Diethylamino) benzaldehyde
EGCG: Epigallocatechin‐3‐gallate
FAE: Fetal Alcohol Effects
FAS: Fetal Alcohol Syndrome
FASD: Fetal Alcohol Spectrum Disorders
GABA: Gamma‐Aminobutyric Acid
GGTP: γ‐glutamyl transpeptidase
GSH: Glutathione
IGF: Insulin‐like Growth Factor
IGF‐1: Insulin‐like Growth Factor 1
IGF‐2: Insulin‐like Growth Factor 2
IGFBP: Insulin‐like Growth Factor Binding Protein
MDA: Malondialdehyde
MnSOD: Manganese‐dependent Superoxide Dismutase
MRI: Magnetic Resonance Imaging
NAD^+^
: Nicotinamide Adenine Dinucleotide (oxidized)
NGF: Nerve Growth Factor
NMDA: N‐methyl‐D‐aspartate
NOX: NADPH Oxidase
NTDs: Neural tube defects
ODC: Ornithine Decarboxylase
PAE: Prenatal Alcohol Exposure
pFas: Partial FAS
PNEE: Prenatal ethanol exposure
POMC: Proopiomelanocortin
PSG: Polysomnography
RDS: Respiratory Distress Syndrome
ROS: Reactive Oxygen Species
Shh: Sonic Hedgehog
SOD: Superoxide Dismutase
WHO: World Health Organization

## 1 Introduction

### 1.1 General Background

The World Health Organization [1] definitively states that congenital anomalies (also known as birth defects) are structural or functional abnormalities that occur during intrauterine life and can be detected in the uterus, at birth, or at a later point in life. These problems can affect different parts of the body and range from minor defects to severe conditions that can result in infant mortality [2]. They result from genetic mutations, chromosomal abnormalities, environmental exposures, maternal infections, or a combination of such factors [3]. Furthermore, congenital anomalies are a leading cause of infant morbidity and mortality worldwide, especially in low‐ and middle‐income countries, where early detection and intervention may be limited [1]. Preventive strategies are clear: ensure adequate maternal nutrition, avoid teratogens, take folic acid supplements, and ensure access to effective prenatal care [4].

Alcohol, specifically ethanol, is a small, water‐soluble organic compound that exerts central nervous system (CNS) depressant effects and is widely consumed for recreational purposes across cultures [5]. Ethanol's primary action is to enhance the inhibitory function of the neurotransmitter gamma‐aminobutyric acid (GABA) and to alter neuronal membrane fluidity [6]. Ethanol is a toxic substance with well‐documented teratogenic, hepatotoxic, and neurotoxic effects, particularly when consumed excessively or chronically [7, 8]. Its hepatic metabolism generates reactive oxygen species (ROS) and acetaldehyde, both of which cause cellular damage and oxidative stress [9]. Despite these risks, alcohol remains one of the leading preventable causes of disease worldwide [5].

Alcohol is a well‐recognized teratogen capable of inducing a broad spectrum of physical and behavioral effects in the developing fetus. One of the most severe outcomes is fetal alcohol syndrome (FAS) [10]. FAS represents the most severe manifestation of fetal alcohol spectrum disorders (FASDs), a group of conditions resulting from prenatal alcohol exposure (PAE), and is characterized by growth retardation, facial dysmorphology, and CNS abnormalities. Notably, FAS is the leading preventable cause of neurodevelopmental disorders and intellectual disabilities. Ethanol readily crosses the placenta and disrupts fetal development, particularly affecting the brain and craniofacial structures, leading to irreversible deficits that may persist throughout life. Nevertheless, FAS remains frequently underdiagnosed and underrecognized in both clinical practice and public health policy [11].

Animal and human studies have consistently demonstrated that ethanol readily crosses the placenta and diffuses into the fetal compartment [12]. Clinical observations and experimental research have shown that maternal alcohol consumption during pregnancy can result in characteristic facial features, pre‐ and postnatal growth restriction, and CNS dysfunction in offspring [13, 14], which were later recognized as hallmark features of FAS. However, findings are not entirely uniform across populations; for example, a large‐scale Japanese cohort study reported that low‐to‐moderate maternal alcohol consumption during pregnancy was not significantly associated with major congenital malformations, including severe brain abnormalities [15]. Over the past four decades, FAS has been increasingly defined as a constellation of birth defects encompassing growth restriction, craniofacial anomalies, intellectual disabilities, and lifelong neurological and behavioral impairments associated with PAE [16, 17, 18, 19].

In summary, this review explores the histopathological impact of maternal alcohol consumption on the placenta and major fetal organs such as the brain, heart, liver, and kidneys in the context of current scientific literature. Despite the extensive body of research on FAS and FASDs, existing reviews predominantly emphasize epidemiology, diagnostic criteria, clinical phenotypes, or isolated molecular mechanisms, often without integrating these findings at the level of tissue and organ pathology. Consequently, the structural and histopathological consequences of PAE across different fetal organs remain fragmented, under‐synthesized, and insufficiently contextualized within a unified developmental framework. In response to this gap, the present narrative review adopts a histopathology‐driven, organ‐specific analytical framework to systematically integrate experimental and clinical evidence on PAE induced alterations across multiple fetal organ systems, including the placenta, brain, cardiovascular system, liver, kidneys, lungs, visual system, and limbs. By synthesizing structural, cellular, and tissue level changes and aligning these morphological findings with their underlying molecular and cellular mechanisms, this review aims to identify convergent pathological patterns, shared vulnerability pathways, and organ dependent susceptibilities. Through this integrative perspective, the review advances a cohesive understanding of ethanol teratogenicity as a multi‐organ pathological process and seeks to bridge the gap between mechanistic research and clinically relevant structural outcomes.

### 1.2 Epidemiology, Etiology, and Diagnostic Features of FAS

Alcoholic beverages contain ethanol, a psychoactive and toxic substance capable of inducing dependence. In 2019, approximately 2.6 million deaths worldwide were attributed to alcohol consumption [20]. To determine how often women of childbearing age drink alcohol, including those World Health Organization were pregnant or could become pregnant, the CDC analyzed data from the 2002 Behavioral Risk Factor Surveillance System survey. The analysis revealed that approximately 10% of pregnant women consumed alcohol, and about 2% engaged in heavy or frequent drinking. Moreover, more than half of women not using contraception (and thus potentially at risk of pregnancy) reported alcohol consumption, with 12.4% engaging in excessive use [21]. In another study involving 4088 women in the United States, it was found that 30.3% consumed alcohol during pregnancy, and 8.3% engaged in binge drinking. Although alcohol consumption declined after the first month of pregnancy, 2.7% continued to drink throughout all trimesters. Prepregnancy binge drinking was identified as the most significant predictor of alcohol use during pregnancy. Other associated risk factors included smoking, unplanned pregnancies, and White ethnicity [22]. Excessive alcohol intake has been specifically associated with physical and cognitive impairments, including the development of FAS [23]. Historical understanding of FAS provides essential context for its identification and prevention. In 1973, researchers first described the core features of FAS and the risks associated with pre‐pregnancy alcohol use. In 1981, the United States Surgeon General issued a warning regarding alcohol‐related birth defects, which was reiterated in 2004 [21]. FASDs result PAE, and alcohol is considered highly teratogenic to the fetus. Even minimal alcohol consumption at any stage of pregnancy can result in irreversible damage and contribute to FASD [24]. The diagnosis of FAS necessitates the manifestation of three core features: distinctive facial anomalies, growth deficiency, and CNS abnormalities. These features may evolve with age; facial traits often become less distinct during adolescence, though key characteristics generally remain identifiable. Even if growth deficiency is not apparent during evaluation, a history of prenatal growth restriction supports the diagnosis. Growth deficiencies not attributable to nutritional deprivation should also be considered [21]. FASDs encompass a range of structural and neurodevelopmental abnormalities caused by PAE, with five diagnostic categories including FAS, partial FAS, ARND, ARBD, and the now‐outdated term FAE, which has been replaced by more specific terminology. While FAS can be diagnosed without confirmed alcohol exposure, other forms require documented maternal history, which is particularly critical for identifying PAE in asymptomatic newborns. Maternal risk factors such as advanced age, high blood alcohol levels, substance use, cohabitation with an alcohol abuser, low socioeconomic status, prior child with FAS, and social instability increase fetal vulnerability to alcohol‐related outcomes [25].

A comparative study demonstrated significantly higher prevalence and severity of FAS among children from lower socioeconomic backgrounds. While only 4.5% of children born to upper middle‐class alcoholic mothers were diagnosed with FAS, the rate reached 70.9% among those from lower‐class families. Growth retardation, congenital anomalies, and neurodevelopmental disorders, including attention deficits, were considerably more prevalent in the lower‐class group, underscoring the critical influence of socioeconomic factors on FAS outcomes [26]. A cohort study evaluated the impact of PAE on cognitive function using data from 2227 individuals across six United States longitudinal cohorts. Generalized additive models, incorporating drinking frequency and dose per occasion, revealed that total daily alcohol intake alone was not a sufficient predictor. Cognitive impairment was significantly elevated when maternal alcohol consumption exceeded approximately three drinks per occasion, whereas low‐dose exposure (~1 drink/occasion) was not associated with notable effects, regardless of frequency [27]. In a longitudinal neuroimaging cohort study from the European Longitudinal Study of Pregnancy and Childhood dataset, even moderate PAE during mid‐pregnancy, but not preconception, was linked to long‐term alterations in brain reward processing and increased cannabis use in young adulthood. Functional magnetic resonance imaging (MRI) data from 191 participants (aged 28–30 years) performing the Monetary Incentive Delay task demonstrated heightened activation in multiple brain regions associated with PAE. These effects were independent of maternal education, prenatal depression, and current alcohol use, supporting the conclusion that no level of alcohol consumption during pregnancy is safe in terms of long‐term neurodevelopmental outcomes [28]. The teratogenic effects of alcohol are dose‐dependent and more pronounced with high or frequent intake. However, no threshold of alcohol consumption during pregnancy has been deemed safe. Even minimal exposure may pose irreversible risks to fetal development [29].

Understanding the biological mechanisms underlying alcohol's teratogenicity elucidates its severe impact on the fetus. Alcohol crosses the placenta readily, resulting in fetal blood alcohol concentrations similar to those of the mother. Due to the immaturity of the fetal liver and its limited capacity to metabolize alcohol, exposure is prolonged. As a known teratogen, alcohol can disrupt fetal development at any stage, and complete abstinence during pregnancy is strongly recommended [24, 29]. The fetus remains dependent on maternal metabolic processes; however, because alcohol dehydrogenase activity in the fetal liver is less than 10% of adult levels, alcohol persists in the fetal circulation for extended periods [30]. Additionally, the amniotic fluid acts as a reservoir, retaining alcohol and prolonging fetal exposure [31]. Consequently, alcohol consumption during pregnancy induces widespread and lasting toxic effects that can impact virtually every organ system in the developing fetus (Table 1).

**TABLE 1:** Key epidemiological, diagnostic, and risk factors associated with FAS.

Category	Details
Prevalence (United States)	− 10% of pregnant women consume alcohol [21] − 2% heavy/frequent drinking [21] − 30.3% alcohol use during pregnancy [22] − 8.3% binge drinking [22] − 2.7% throughout all trimesters [22]
Risk factors	− Pre‐pregnancy binge drinking [22] − Smoking [22] − White ethnicity [22] − Unplanned pregnancy [22] − Maternal age ≥ 25 [25] − Alcohol/substance abuse [25] − Low socioeconomic status, unemployment, social instability [25] − Prior child with FAS [25]
Diagnostic criteria for FAS	− Characteristic facial anomalies [21] − Growth deficiency [21] − CNS abnormalities [21] − History of prenatal alcohol exposure (PAE) [21]
FASD subtypes	− Fetal Alcohol Syndrome Disease FASDs: subtypes [25] − Fetal Alcohol Syndrome (FAS): Full diagnostic triad (facial dysmorphia, growth deficiency, and CNS abnormalities); can be diagnosed even without confirmed PAE − Partial Fetal Alcohol Syndrome (pFAS): Some facial features of FAS + CNS abnormalities + confirmed alcohol exposure − Alcohol‐Related Neurodevelopmental Disorder (ARND): Neurodevelopmental and behavioral impairments only, no physical features, requires confirmed PAE − Alcohol‐Related Birth Defects (ARBD): Structural malformations (e.g., of the heart, kidneys, or eyes) + confirmed alcohol exposure − Fetal Alcohol Effects (FAE): Historical term, previously used for individuals with some but not all features of Fetal Alcohol Syndrome (FAS); largely replaced by more specific diagnoses
Socioeconomic disparities	− Fetal Alcohol Syndrome (FAS) rate: 70.9% in lower‐class families vs. 4.5% in upper‐middle class [26]
Neurodevelopmental outcomes	− Cognitive impairments linked to ≥ 3 drinks/occasion [27] − Magnetic Resonance Imaging (MRI) studies show altered reward processing from mid‐pregnancy exposure [28]
Biological basis	− Alcohol crosses placenta easily [29] − Fetal liver has < 10% of adult alcohol dehydrogenase (ADH) activity [30] − Amniotic fluid prolongs exposure [31] − Complete abstinence during pregnancy is essential [24, 29]

*Note:* This table summarizes key epidemiological data, diagnostic criteria, risk factors, and biological mechanisms associated with fetal alcohol syndrome (FAS).

Collectively, these epidemiological, etiological, and diagnostic findings indicate that FAS and related spectrum disorders reflect the cumulative outcome of widespread developmental injury rather than isolated functional impairments. The marked variability in clinical presentation, the absence of a safe exposure threshold, and the persistence of neurodevelopmental and systemic effects across the lifespan strongly suggest that PAE disrupts fetal development at the cellular, tissue, and organ levels. Accordingly, a comprehensive understanding of FAS and FASD requires moving beyond population‐level observations to examine the underlying histopathological alterations that mediate these clinical phenotypes. This perspective provides the basis for the organ‐specific analysis of ethanol‐induced structural and cellular damage presented in the subsequent sections.

## 2 Materials and Methods

This narrative review was designed to comprehensively evaluate the organ‐specific histopathological effects of PAE on fetal development. The review focused explicitly on tissue‐level, cellular, and morphological alterations rather than purely behavioral, epidemiological, or postnatal outcomes.

### 2.1 Literature Search Strategy

A structured and reproducible literature search was conducted using the PubMed and Google Scholar databases between April and June 2025, covering publications from the late 1960s through June 2025. Search terms were defined prior to screening and combined using Boolean operators to ensure consistency across all organ systems. Core alcohol‐related terms included *fetal alcohol syndrome*, *fetal alcohol spectrum disorders*, *prenatal alcohol exposure*, *ethanol teratogenicity*, *maternal alcohol consumption*, *alcohol and pregnancy*, *histopathology*, and *morphology*.

To capture organ‐specific evidence, these core terms were systematically combined with keywords related to individual fetal organs and systems. For the nervous system, search combinations targeted the brain, neural tube, and neural crest (e.g., *prenatal alcohol exposure AND brain AND histopathology*; *fetal alcohol syndrome AND neurodevelopment*; *ethanol teratogenicity AND neural tube*; *prenatal alcohol exposure AND neural crest*). Placental searches incorporated terms related to placental development and pathology (e.g., *prenatal alcohol exposure AND placenta AND histopathology*; *ethanol AND placental pathology*; *maternal alcohol consumption AND trophoblast*). Cardiovascular searches focused on heart development and congenital anomalies (e.g., *prenatal alcohol exposure AND heart development*; *ethanol teratogenicity AND cardiogenesis*; *fetal alcohol syndrome AND congenital heart defects*).

Additional searches addressed liver pathology and hepatic development (e.g., *prenatal alcohol exposure AND liver pathology*; *ethanol AND fetal liver development*), renal histology and nephrogenesis (e.g., *prenatal alcohol exposure AND kidney histology*; *ethanol teratogenicity AND nephrogenesis*), pulmonary development and lung histopathology (e.g., *prenatal alcohol exposure AND lung development*; *ethanol AND fetal lung histopathology*), and ocular and visual system development (e.g., *fetal alcohol syndrome AND ocular development*; *prenatal alcohol exposure AND optic nerve*).

In parallel, mechanistic searches were performed to identify studies examining the biological pathways underlying ethanol‐induced developmental injury. These included combinations such as *prenatal alcohol exposure AND oxidative stress*, *prenatal alcohol exposure AND apoptosis*, *ethanol teratogenicity AND inflammation*, *prenatal alcohol exposure AND angiogenesis*, and *ethanol AND retinoic acid signaling*, ensuring comprehensive coverage of the cellular and molecular mechanisms contributing to organ‐specific histopathological alterations associated with PAE.

During the screening process, a limited number of studies describing endocrine/metabolic alterations and epigenetic or long‐term functional outcomes were also encountered within the scope of the primary histopathology‐oriented search strategy. These topics were not targeted using separate or dedicated search terms and therefore were not considered primary endpoints of this review. Accordingly, they are presented as secondary and supportive consequences of PAE rather than organ‐specific histopathological findings and are summarized under the “Other Impacts” subsection in the Results section.

### 2.2 Eligibility Criteria

Eligibility criteria were defined prior to the screening process and were applied consistently at all stages of study selection.

### 2.3 Inclusion Criteria

Studies were considered eligible if they addressed PAE occurring during embryonic or fetal development and reported organ‐specific histopathological, morphological, or structural alterations at the cellular, tissue, or organ level. Histopathological relevance was applied as a primary inclusion criterion during title/abstract screening and was further confirmed during full‐text evaluatioEligible studies examined at least one relevant fetal organ or system, including the placenta, brain (including neural tube and neural crest derivatives), cardiovascular system, liver, kidney, lung, limbs, or visual system. Both primary studies (human clinical studies, animal experiments, or in vitro models) and review articles were included, provided that they explicitly synthesized organ‐specific histopathological findings relevant to PAE. Only English‐language publications were considered.

### 2.4 Exclusion Criteria

Studies were excluded if they involved a wrong population, defined as alcohol exposure occurring postnatally, during adolescence, or in adulthood, or if prenatal exposure could not be clearly distinguished. Articles were also excluded for wrong outcomes, namely when reported findings were limited to behavioral, cognitive, psychiatric, epidemiological, or psychosocial outcomes without accompanying histological, morphological, or tissue‐level data. Studies evaluating toxic agents other than ethanol or combined exposures in which ethanol‐specific effects could not be isolated were excluded. Additional exclusion criteria included publication type (commentaries, editorials, conference abstracts, or opinion pieces lacking original or integrative histopathological content), wrong model (nonmammalian species without demonstrated relevance to mammalian fetal histopathology), and language restriction (non‐English publications).

### 2.5 Study Selection Process

The database search identified 1213 records through PubMed and 500 additional records through other sources (Google Scholar), resulting in a total of 1713 records. After removal of 87 duplicates, 1626 unique records remained. Following title and abstract screening, 1481 articles were excluded because they did not meet the predefined eligibility criteria, most commonly due to the absence of organ‐specific histopathological outcomes.

**FIGURE 1:**
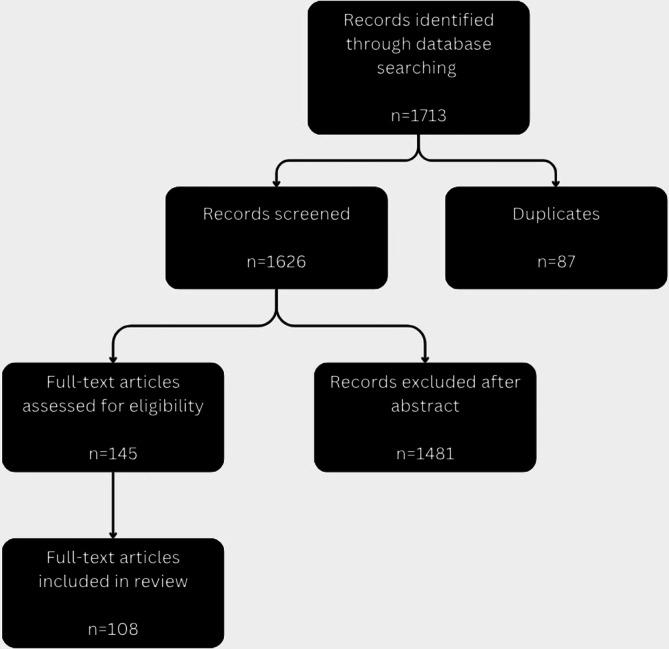
Flow diagram of study selection (created with Canva by the corresponding author).

Full texts of 145 articles were assessed for eligibility. Of these, 108 studies were included in the final narrative synthesis because they reported primary or integrative organ‐specific histopathological findings derived from experimental animal models or clinical studies. 37 full‐text articles were excluded at this stage because, despite addressing PAE, they lacked sufficient tissue‐level or organ‐specific histopathological data to support the objectives of this review (Figure 1).

### 2.6 Methodological Transparency

This study is a narrative review, although the selection process was illustrated using the PRISMA 2020 flow diagram to enhance transparency. No PROSPERO registration or formal risk‐of‐bias assessment was conducted; therefore, the review does not fulfill the methodological criteria of a systematic review. All schematic figures were created using BioRender (https://biorender.com) and Canva, and all tables were prepared using Microsoft Word.

## 3 Results

This narrative review summarizes organ‐specific histopathological findings associated with PAE across major fetal organs and systems. The included studies comprise experimental animal models, human clinical observations, and postmortem analyses, providing an integrated overview of tissue‐level alterations induced by ethanol exposure during embryonic and fetal development. The results are organized by organ system to highlight common pathological patterns, organ‐specific susceptibilities, and variability related to exposure timing, dosage, and experimental or clinical context. Where relevant, discrepancies among studies are addressed in relation to differences in study design, species, and developmental stage at exposure.

### 3.1 Pathophysiological Mechanisms in FAS

FAS arises from PAE that disrupts key developmental processes such as cell proliferation, migration, and differentiation [32]. Central mechanisms include oxidative stress, apoptosis, and mitochondrial dysfunction [32, 33], which particularly affect the developing brain and placenta, leading to structural and functional abnormalities [34, 35]. To better understand how these effects unfold, it is important to consider the role of oxidative stress in fetal development. Reactive oxygen species (ROS) are naturally occurring, unstable molecules that play essential roles in normal development, including superoxide anions, hydrogen peroxide, and hydroxyl radicals. However, ROS levels can become elevated due to in utero exposure to environmental toxins or pharmaceuticals, resulting in oxidative stress [36]. Ethanol exposure during prenatal development has been shown to increase oxidative stress in various developing organs, particularly the brain [32]. Taken together, these findings indicate that oxidative stress is not merely a downstream consequence of prenatal ethanol exposure, but a central early mechanism linking ethanol metabolism to widespread organ‐specific histopathological injury.

Chronic ethanol exposure during the third‐trimester equivalent of pregnancy induces oxidative stress in the fetus through activation of the hypothalamic–pituitary–adrenal axis and enhanced ROS generation via CYP2E1‐mediated metabolism. Due to immature antioxidant defenses, the fetus is particularly susceptible to oxidative damage, leading to lipid, protein, and DNA peroxidation as well as apoptosis. Chronic ethanol exposure also reduces glutathione (GSH) levels in the fetal liver and brain, while early CYP2E1 expression in the fetal liver and placenta may contribute to teratogenicity via toxic ethanol metabolites [33]. Importantly, chronic ethanol exposure enhances CYP2E1 activity in both microsomal and mitochondrial fractions of the fetal liver, supporting a critical role for mitochondrial dysfunction and ROS overproduction in ethanol‐induced fetal injury [33]. Across experimental models, the severity of oxidative injury consistently varies with developmental stage, tissue‐specific metabolic demand, and antioxidant capacity, providing a mechanistic explanation for differential organ vulnerability.

Ethanol is primarily metabolized to acetaldehyde by alcohol dehydrogenase (ADH) in the liver and by catalase in the brain, followed by conversion to acetate by aldehyde dehydrogenase 2 (ALDH2) in both organs. While peripheral acetaldehyde accumulation induces aversion to ethanol, its production in the brain's ventral tegmental area enhances ethanol's rewarding effects, indicating a dual, site‐specific role of acetaldehyde in modulating ethanol consumption [37]. Due to reduced alcohol clearance and elevated oxidative stress, ethanol‐exposed fetuses are particularly susceptible to metabolic and epigenetic disturbances [38].

Prenatal ethanol exposure markedly enhances ROS production in the developing brain through ethanol metabolism by enzymes such as ADH and CYP2E1, resulting in the accumulation of ROS and toxic intermediates such as acetaldehyde. The fetal brain is especially vulnerable due to its high oxygen consumption, abundance of unsaturated fatty acids, and limited antioxidant defenses, including low levels of GSH and superoxide dismutase (SOD). This imbalance promotes oxidative damage to lipids, proteins, and DNA, contributing to the neurodevelopmental deficits observed in FASD [32]. Similarly, PAE significantly reduces GSH levels in developing liver and brain tissues, increasing fetal susceptibility to alcohol‐induced toxicity and free radical damage [39].

Experimental studies provide direct evidence supporting these mechanisms. Prenatal ethanol exposure significantly increases the expression and activity of NADPH oxidase (NOX), particularly its catalytic and regulatory subunits, in gestational Day 9 mouse embryos, resulting in elevated ROS production, oxidative DNA damage, and caspase‐3‐mediated apoptosis. Co‐treatment with the NOX inhibitor diphenyleneiodonium markedly attenuated these effects, highlighting the pivotal role of NOX‐derived ROS in ethanol‐induced oxidative stress and embryotoxicity [40]. Although individual studies emphasize different molecular contributors, their convergence on oxidative DNA damage and apoptosis underscores a shared pathogenic trajectory.

Beyond oxidative stress, ethanol exposure interferes with additional critical signaling pathways. Ethanol disrupts retinoic acid signaling, which is essential for neural tube closure and craniofacial development [41], and activates microglia and astrocytes, inducing neuroinflammation and long‐term disturbances in central nervous system development [42]. Although most FAS studies focus on the brain, placental pathology also plays a crucial role. In ethanol‐exposed mice, placental tissue exhibits vacuolization, lymphocytic infiltration, reduced glycogen and mucopolysaccharide levels, and degeneration of glycogen cells in the basal zone. The labyrinthine region shows altered architecture, cyst formation, fibrinoid accumulation, and reduced fetal vasculature [35]. These placental alterations likely amplify fetal exposure to ethanol and its metabolites, thereby exacerbating injury in highly sensitive organs such as the brain.

In addition to structural and metabolic abnormalities, ethanol affects neurotransmitter systems by readily crossing the placenta and disrupting fetal central nervous system development through apoptosis and necrosis. Ethanol alters oxidative balance, insulin‐like growth factor signaling, glial function, serotonin and glutamate neurotransmission, and glucose metabolism, particularly affecting the basal ganglia, corpus callosum, cerebellum, and hippocampus. These disruptions contribute to impairments in motor coordination, cognition, and executive function, with genetic susceptibility and maternal health further modulating severity [25].

Ethanol exposure during postnatal Days 4–9 results in microencephaly and reduced brain and body weight in neonatal rats. While NMDA receptor subunits remain unchanged, AMPA receptor subunits are significantly reduced, indicating selective impairment of excitatory synaptic signaling in the developing neocortex [34]. While study‐specific differences exist in molecular targets and affected pathways, these findings collectively point toward impaired neuronal survival, migration, and synaptic maturation as shared downstream consequences of prenatal ethanol exposure.

**FIGURE 2:**
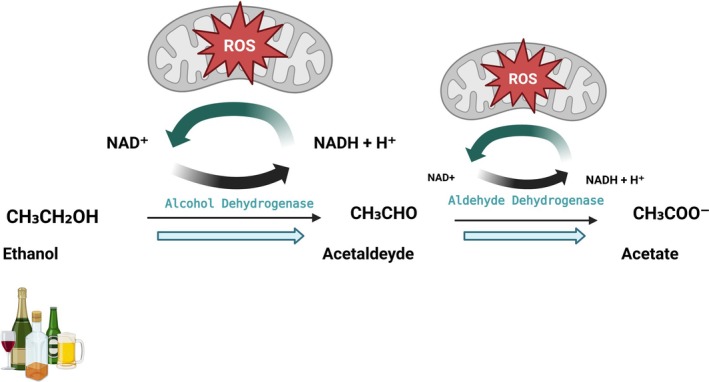
Ethanol metabolism and reactive oxygen species (ROS) generation during pregnancy. Ethanol (CH_3_CH_2_OH) is metabolized to acetaldehyde (CH_3_CHO) by ADH and subsequently to acetate (CH_3_COO^−^) by ALDH. These reactions require NAD^+^ as a coenzyme and result in the generation of NADH and protons (H^+^). Both metabolic steps, particularly within mitochondria, contribute to ROS production, which plays a key role in fetal alcohol syndrome (FAS) pathogenesis. The immature fetal liver and limited enzymatic capacity further prolong exposure and enhance oxidative damage (Created with BioRender.com by corresponding author).

**FIGURE 3:**
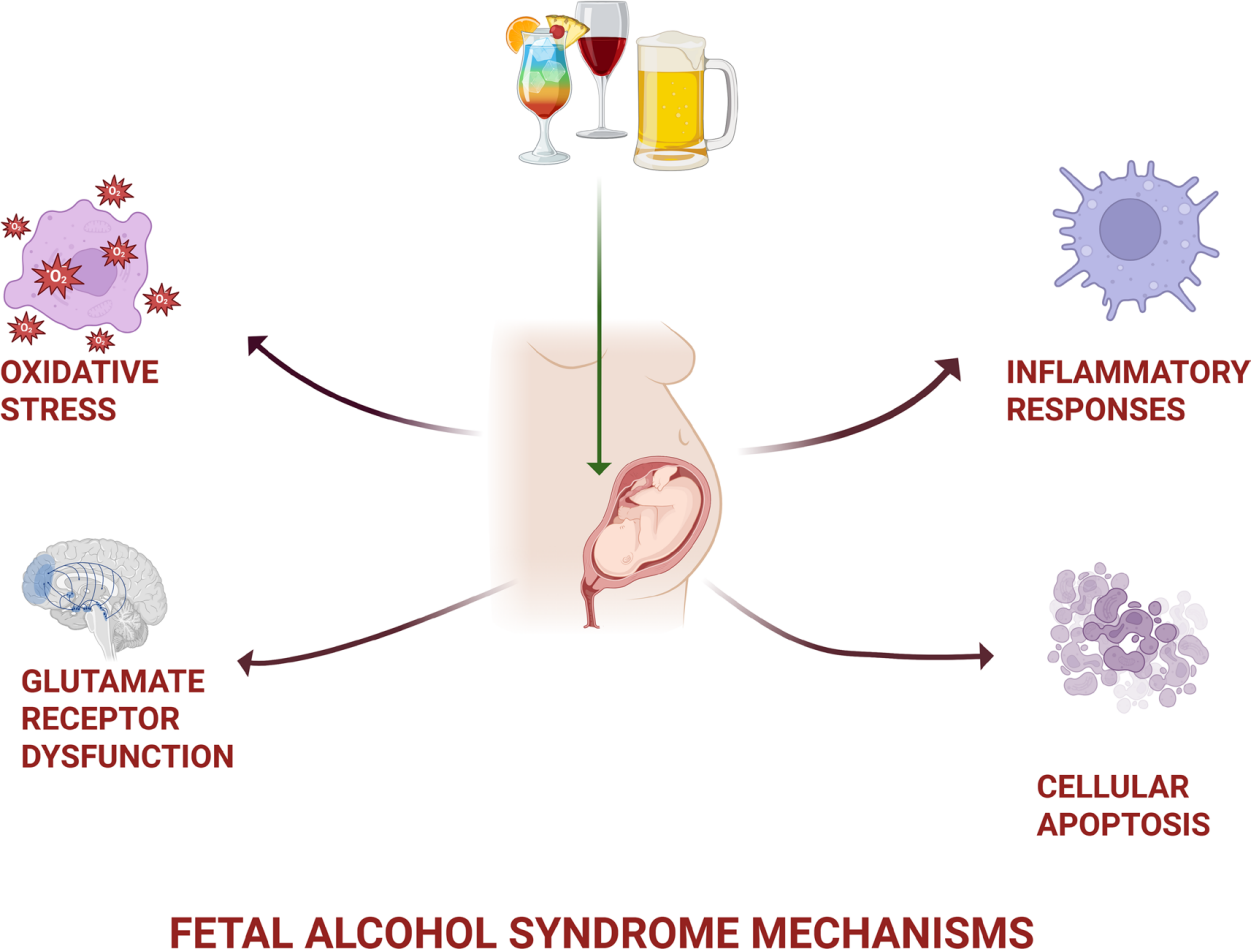
Mechanisms involved in the pathophysiology of fetal alcohol syndrome (FAS). PAE induces oxidative stress, glutamate receptor dysfunction, inflammatory responses, and cellular apoptosis. These interconnected mechanisms contribute to structural and functional abnormalities in the developing fetus, particularly affecting the brain and other organ systems (Created with BioRender.com by corresponding author).

Altogether, these findings demonstrate that the pathogenesis of FAS is not merely multifactorial, but hierarchically organized around early oxidative injury, disrupted signaling pathways, and impaired cellular resilience, which together drive organ‐specific histopathological outcomes during critical windows of fetal development (Table 2, Figures 2 and 3).

**TABLE 2:** Key pathophysiological mechanisms in fetal alcohol syndrome (FAS).

Main mechanism	Pathological features
Oxidative stress	Ethanol metabolism via alcohol dehydrogenase (ADH) and cytochrome P450 2E1 (CYP2E1) increases reactive oxygen species (ROS), including superoxide, hydrogen peroxide, and hydroxyl radicals [32, 33]. The fetal brain, with low glutathione (GSH) and superoxide dismutase (SOD), is highly vulnerable [38, 39]. Elevated NADPH oxidase (NOX) activity further amplifies ROS and caspase‐3‐mediated apoptosis, which is mitigated by NOX inhibition [40]
Glutamate receptor dysfunction	Ethanol exposure selectively reduces α‐amino‐3‐hydroxy‐5‐methyl‐4‐isoxazolepropionic acid (AMPA) receptor subunits GluR1 and GluR2/4 in the developing neocortex, while N‐methyl‐D‐aspartate (NMDA) receptor subunits remain unaffected. This impairs excitatory neurotransmission and contributes to microencephaly and neurodevelopmental deficits [34]
Cellular apoptosis	Ethanol exposure induces caspase‐3‐mediated apoptosis and oxidative DNA damage in fetal tissues. NADPH oxidase (NOX) activation contributes to this process, and inhibition with diphenyleneiodonium significantly reduces apoptosis, underscoring the role of NOX‐derived reactive oxygen species (ROS) in ethanol‐induced embryotoxicity [40]
Inflammatory responses	Prenatal alcohol exposure (PAE) activates microglia and astrocytes, leading to increased secretion of proinflammatory cytokines such as TNF‐α and IL‐1β, which interfere with normal central nervous system (CNS) development [42]. Chronic neuroinflammation contributes to long‐term cognitive and behavioral deficits in affected offspring [25]

*Note:* This table summarizes the major mechanisms implicated in FAS pathogenesis, including oxidative stress, glutamate receptor dysfunction, apoptosis, and inflammation. Each mechanism is associated with specific pathological features observed in experimental models and clinical data. All abbreviations are defined below. Referenced studies provide supporting evidence for each feature described.

### 3.2 Organ Specific Effects

#### 3.2.1 Placenta

The placenta has been increasingly recognized as a key structure involved in the pathophysiological effects of PAE, with a growing body of evidence indicating its potential contribution to the development of FASDs. Research has indicated that children from different ethnic groups born to mothers with a chronic alcohol problem show similar patterns of defects in the head, face, limbs, and heart, along with growth deficiency and developmental delay during the prenatal period [13]. These convergent phenotypic patterns across diverse populations suggest that shared biological mechanisms, rather than ethnic or sociocultural factors alone, underlie ethanol‐induced fetal injury, pointing to the placenta as a critical mediating interface. Supporting this, a study reported the consistent presence of placental abnormalities in FAS, indicating a strong association between placental dysfunction and the pathogenesis of FAS [35]. In a prospective birth cohort study conducted in Cape Town, the impact of PAE on the composition of placental cells and the expression of their genes was examined. Alcohol exposure was associated with an increased proportion of Hofbauer cells and significant changes in inflammation‐related gene expression, suggesting that placental inflammation may contribute to the pathogenesis of FASD [43]. Importantly, these findings imply that inflammatory alterations within the placenta are not merely secondary phenomena, but may actively modify the intrauterine environment, thereby amplifying fetal vulnerability to ethanol and its toxic metabolites. In an experimental study, ethanol was administered before and during gestation to evaluate placental growth in rats. Histological examination of placentas collected in late pregnancy revealed that alcohol exposure significantly increased placental weight compared to the control group, particularly during the later stages of gestation. In the alcohol‐exposed group, giant cells were more numerous and larger in size, trophoblastic cells appeared enlarged, and maternal blood vessels in the labyrinth region were markedly dilated and congested with blood cells. These findings suggest that the observed placental enlargement may result from blood cell stagnation within maternal vessels and the expansion of giant and trophoblastic cells in the basal zone [44]. Rather than reflecting enhanced placental growth or function, this increase in placental weight likely represents a maladaptive or compensatory response associated with impaired placental circulation and cellular stress. In contrast, another study exposed pregnant mice to alcohol‐containing solutions corresponding to mild, moderate, and severe exposure levels based on blood alcohol concentrations. Although significant fetal growth disturbances were observed, no increase in fetal mortality or gross structural malformations was detected. Alcohol exposure was associated with a dose‐dependent reduction in placental weight. Histological analysis demonstrated increased intravascular coagulation of maternal erythrocytes within the labyrinth region, accompanied by extensive degenerative changes in the basal zone. These findings indicate that maternal alcohol consumption disrupts both the vascular and endocrine functions of the placenta [45] (Table 3). Taken together, the contrasting placental weight outcomes reported by Eguchi et al. and Kennedy likely reflect differences in ethanol dose, duration, and developmental timing of exposure. These findings underscore the context‐dependent nature of placental responses to PAE, whereby ethanol may induce either compensatory placental enlargement or growth restriction depending on exposure severity and gestational window. Importantly, these studies indicate that PAE induces complex placental pathology involving vascular, inflammatory, endocrine, and cellular compartments. Such alterations should not be regarded as incidental findings, but rather as central pathogenic contributors that may influence fetal growth restriction, organ‐specific histopathological damage, and the multisystem manifestations of FASD.

**TABLE 3:** Pathophysiological mechanisms and organ‐specific effects of prenatal alcohol exposure (PAE).

System/Organ	Mechanisms of action	Observed effects
Placenta	− Villous immaturity and reduced glycogen content in basal zone [35] − Placental inflammation and increased Hofbauer cells [43] − Enlarged placental weight, dilated maternal vessels, and enlarged giant/trophoblastic cells [44] − Intravascular coagulation and degenerative changes in basal zone [45]	− Impaired vascular and endocrine placental function [45] − Reduced nutrient transfer [43] − Placental enlargement and altered architecture [44] − Low birth weight [35]
Brain	− High oxygen‐metabolic rate vulnerability [46] − Ethanol‐induced neuronal and glial dysfunction with neuroinflammation [42] − Acute apoptosis in neurons and oligodendrocytes [47, 48] − Purkinje cell loss [49] − Structural brain changes including microcephaly, basal ganglia volume loss, and hippocampal sparing [50] − Hippocampal neuron loss, reduced synaptic plasticity, and abnormal electrical activity [51] − Mitochondrial glutathione (GSH) depletion and oxidative damage in hippocampus [52] − Subarachnoid hemorrhage risk without white matter loss [53] − Disruption of astrocyte‐oligodendrocyte‐microglia interactions [54] − Impaired fetal habituation to repeated stimuli [55] − Cumulative spatial learning deficits from multi‐stage exposure [56] − Reduced Brain‐derived neurotrophic factor (BDNF) and nerve growth factor (NGF) with cholinergic deficits, partially mitigated by red wine antioxidants [57] − BDNF–TrkB signaling disruption [58] − Neuroprotection by activity‐dependent neurotrophic factor‐12 (ADNF‐12) against ethanol‐induced developmental delays [59] − Oxidative stress‐induced apoptosis blocked by antioxidants [60] − Silybin protection against oxidative damage and fetal mortality [61] − Persistent hypothalamic oxidative stress altering proopiomelanocortin (POMC) expression [62] − Reduced glutathione (GSH) and increased lipid peroxidation reversed by omega‐3 supplementation [63] − Brain catalase‐mediated ethanol metabolism producing acetaldehyde [64]	− Microcephaly [50] − Cortical thinning [50] − Synaptic loss [34]
Neural tube	− Disruption of retinoic acid signaling, abnormal neural gene expression, increased neural progenitor proliferation, and neural plate expansion [41] − Anterior neural plate cell death leading to exencephaly, arhinencephaly, pituitary dysplasia, maxillary hypoplasia, cleft lip [65] − Suppression of Sonic Hedgehog signaling and primary cilia disruption [66] − 28%–40% serotonergic neuron loss in brainstem regions, prevented by ipsapirone [67] − Ethanol‐induced apoptosis in rhombencephalic and serotonergic neurons prevented by S100B via PI3K/pAkt activation [68] − Impaired neuronal migration with altered Ca^2+^, cGMP, and cAMP signaling [69]	− Neural tube defects [41] − Craniofacial malformations [70] − Microcephaly [71] − Abnormal serotonergic neuron density [66]
Neural crest	− Stage‐specific cranial neural crest cell loss causing variable craniofacial malformations [70] − Apoptosis and elevated homocysteine in migrating neural crest cells, prevented by 5‐methyltetrahydrofolate [71]	
Visual system	− Disruption of retinal and optic nerve development during early embryogenesis [72] − Impairment of neural crest migration affecting ocular structures [72] − Teratogenic effects during critical periods of ocular morphogenesis, including anterior segment development [73] − Central nervous system (CNS) injury affecting visuospatial processing and working memory, reflected in abnormal saccadic eye movement control [74]	− Microphthalmia, microcornea, coloboma, cataract, blepharoptosis, persistent hyperplastic primary vitreous, nystagmus, anterior segment dysgenesis such as Peter's and Axenfeld's anomalies [73, 75] − Fundus anomalies, particularly optic nerve hypoplasia, and strabismus, often persistent despite interventions [75] − Severe visual impairment in some cases despite stable ocular malformations [75] − Deficits in visuospatial processing are correlated with eye movement errors [74]. − High specificity of the fetal alcohol syndrome disorder (FASD) Eye Code for detecting ocular abnormalities [76]
Cardiovascular system	− Disruption of Wnt/β‐catenin signaling during gastrulation, leading to myocardial wall and valve defects [77] − Induction of oxidative stress and cardiac neural crest cell death [78, 79] − Impairment of angiogenesis and vascular remodeling (loss of radial microvascular organization) [80] − Endothelial and smooth muscle dysfunction, increased arterial stiffness [53] − Activation of endocannabinoid receptors (particularly CB2) altering vascular tone [81, 82] − Genetic susceptibility influencing myocardial maturation and structural outcomes [78]	− Structural heart defects: atrial septal defect, ventricular septal defect tetralogy of Fallot, great vessel anomalies [83] − Myocardial wall abnormalities, valve malformations, endocardial cushion defects [77, 84] − Ventricular thinning, chamber dilation, reduced myosin content [78] − Dysplastic pulmonary arteries associated with ventricular septal defect [85] − Reduced cortical vascular density and collateral vessel formation [80] − Decreased fetal abdominal and head circumferences (no femur length change) [86] − Transient decreases in systolic velocity of anterior and middle cerebral arteries during intoxication (resolved over time) [86] − Vasodilation of fetal cerebral arteries via cannabinoid receptor‐mediated pathways (mid‐gestation only; absent near term) [81, 82] − Postnatal persistence of vascular stiffness, increasing long‐term cardiovascular disease risk [53]
Liver	− Disruption of glycogen metabolism via decreased glycogen synthase and phosphorylase activity [87] − Inhibition of hepatocyte proliferation through reduced DNA synthesis without affecting growth factor expression [88] − Oxidative stress and altered antioxidant enzyme activity, including decreased superoxide dismutase (SOD) activity [89] − Induction of hepatic steatosis and fibrosis through collagen deposition and Ito cell activation [90] − Disruption of insulin/IGF‐1 and Akt/PRAS40 signaling pathways, mitigated by soy protein supplementation [91, 92] − Altered iron metabolism with increased hepatic iron accumulation and elevated hepcidin expression [93] − Impaired hepatocyte differentiation and bile duct development [94]	− Fetal growth retardation, hypoglycemia, and reduced hepatic glycogen stores [87] − Decreased liver‐to‐body weight ratio and suppressed DNA synthesis [88] − Increased hepatic enolase and ADH activity with reductions in other liver enzymes [89] − Parenchymal steatosis, portal and perisinusoidal fibrosis with myofibroblast proliferation [90] − Prevention of fetal loss, placental dysfunction, and signaling deficits by dietary soy substitution [91, 92] − Increased fetal hepatic iron with concurrent reduction in brain iron and iron transport proteins [93] − Reduced liver and bile duct size, decreased hepatocyte number, and impaired differentiation in zebrafish model [94]
Lung	− Oxidative stress with decreased superoxide dismutase (SOD) activity, reduced GSH, and increased malondialdehyde (MDA) levels, leading to cellular injury in lung tissue [95] − Impaired surfactant production and altered surfactant protein expression [53, 96] − Increased collagen deposition in fetal lung tissue [53, 96]. − Impaired cellular proliferation and growth restriction in lung parenchyma [97]. − Possible acceleration of fetal lung maturation [98]. − Increased vulnerability to respiratory infections and aspiration [99]	− Marked histological damage in lung tissue, more severe than in brain tissue [95]. − Reduced surfactant protein A and B expression, altered phospholipid composition, decreased lung compliance, and impaired innate immune responses; persistent elevation of Surfactant protein D mRNA postnatally [53, 96]. − Lung fibrosis‐like changes and decreased elasticity, partially reversible after birth [53, 96]. − Reduced lung weight, DNA, RNA, and protein content; lung hypoplasia with fewer and smaller cells [97]. − Decreased incidence of respiratory distress syndrome (RDS) in preterm infants (< 37 weeks) born to mothers who consumed alcohol [98]. − Severe aspiration pneumonia in a 6‐month‐old infant with FAS, along with postnatal growth retardation, facial anomalies, and brain abnormalities [99]
Kidney	− Disruption of renal morphogenesis, leading to congenital anomalies such as hydronephrosis [100] − Reduction in total nephron number, potentially predisposed to hypertension and renal disease later in life [53] − Impairment of distal tubular acidification and potassium excretion due to tubular transport defects [101] − Defective urinary concentration and acidification capacity, suggesting persistent subclinical tubular dysfunction [102]	− High incidence of hydronephrosis [100] − Decreased nephron number without overt renal injury at birth [53] − Elevated minimum urine pH, reduced net acid and potassium excretion [101] − Impaired maximal urinary concentration after water deprivation and vasopressin, defective acidification after ammonium chloride loading [102]
Limbs	− Early gestational prenatal alcohol exposure (PAE) exerts systemic teratogenic effects, disrupting normal morphogenesis across multiple organ systems, likely through interference with cell proliferation, differentiation, and patterning processes during critical stages of limb bud and organ development [103]	− Dose‐related increase in congenital malformations involving skeletal, neurological, urogenital, and cardiovascular systems; limb defects such as adactyly, ectrodactyly, and syndactyly; ocular malformations including anophthalmia and microphthalmia [103]

*Note:* This table summarizes the pathophysiological mechanisms and organ‐specific histopathological effects associated with PAE. For each system or organ, key molecular and cellular mechanisms are listed alongside the observed morphological and functional outcomes, supported by relevant experimental and clinical studies.

#### 3.2.2 Nervous System

##### 3.2.2.1 Brain

The brain is one of the most vulnerable organs to PAE, as ethanol disrupts both structural and functional development across multiple neural systems. This heightened vulnerability is partly explained by the brain's exceptionally high oxygen metabolic rate, with neural cells consuming nearly 20% of the body's total oxygen [46]. This substantial metabolic demand, together with immature antioxidant defenses during development, creates a permissive environment for ethanol‐induced oxidative injury. The mechanisms underlying ethanol's detrimental effects on the developing nervous system are multifactorial. Ethanol can directly alter neuronal function in the developing central nervous system (CNS) and also affects glial cells, including microglia and astrocytes, which are essential for maintaining neural homeostasis. Although these cells serve as resident immune components of the brain, excessive or dysregulated glial activation may damage CNS cells and result in abnormal neural functioning. Accordingly, ethanol exposure during brain development has been associated with glial activation and neuroinflammation, contributing to FASD‐related pathology [42].

Evidence from experimental models consistently demonstrates that ethanol exposure rapidly triggers apoptotic pathways in the developing brain. A single exposure of the fetal nonhuman primate brain to alcohol during early or late third‐trimester–equivalent pregnancy causes extensive acute apoptotic cell death in both gray and white matter, with dying gray matter cells identified as neurons and affected white matter cells belonging to the oligodendrocyte lineage [47]. Similarly, ethanol exposure during the synaptogenesis period in infant rats and mice induces widespread apoptotic neurodegeneration, affecting Purkinje cells in the cerebellum, deep cerebellar nuclei, and related brainstem regions such as the nucleus pontis and inferior olivary complex [48]. Together, these findings identify synaptogenesis as a particularly sensitive developmental window for ethanol‐induced neuronal loss. Intervention‐based studies further indicate that the timing of exposure is critical for neuroprotection. One study assessed the potential of melatonin to reduce alcohol‐induced oxidative damage in newborn rats by examining cerebellar Purkinje cell loss. Alcohol exposure between postnatal Days 4 and 9 caused significant Purkinje cell loss in the vermis and lobule I, with the most severe damage observed in lobule I. However, melatonin administration alone had no effect on neuronal counts and failed to attenuate alcohol‐induced cell loss [49]. This lack of efficacy suggests that antioxidant supplementation may be insufficient once apoptotic cascades have been initiated during critical developmental periods. Human neuroimaging and behavioral studies mirror these experimental observations and reveal region‐specific vulnerability. Children prenatally exposed to alcohol have been shown to exhibit microcephaly, reduced basal ganglia volume, and pronounced white matter loss, particularly in the parietal lobes, while hippocampal volume appears relatively preserved [50]. PAE impairs cognitive functions such as learning, memory, and attention by disrupting hippocampal development. Correspondingly, animal studies demonstrate neuronal loss, reduced synaptic plasticity, and abnormal electrical activity in the hippocampus, with additional involvement of the frontal cortex and cerebellum [51]. At the subcellular level, mitochondrial dysfunction emerges as a key mediator of ethanol‐induced brain injury. In a study examining chronic prenatal ethanol exposure in guinea pigs, ethanol‐exposed offspring exhibited reduced brain and hippocampal weights. Postnatally, mitochondrial glutathione (GSH) levels in the hippocampus were significantly decreased, whereas cytosolic GSH and lipid peroxidation markers remained unchanged. These findings indicate selective mitochondrial GSH depletion, implicating mitochondrial oxidative stress as a contributor to hippocampal injury [52]. Not all ethanol‐induced brain alterations manifest as overt neurodegeneration, highlighting variability across exposure paradigms. In a sheep model, prolonged PAE during late gestation did not result in overt white matter damage, gliosis, or apoptosis. Nevertheless, small subarachnoid hemorrhages were observed in a subset of ethanol‐exposed fetuses, suggesting increased susceptibility to cerebrovascular injury even in the absence of persistent parenchymal damage [53]. This observation underscores vascular fragility as an additional component of ethanol‐induced neuropathology. Beyond neurons, ethanol disrupts glial populations and intercellular communication within the developing brain. Intrauterine alcohol exposure affects astrocytes, oligodendrocytes, and microglia, and its impact should be considered not only at the level of individual cell types but also in terms of altered glia–glia and glia–neuron interactions. Consequently, therapeutic strategies for FASD may need to target broader regulatory mechanisms governing cellular communication rather than focusing solely on isolated cell populations [54]. Functional consequences of these structural alterations are evident even before birth. A cohort study examining fetal habituation to repeated stimuli from 35 weeks' gestation demonstrated that fetuses of mothers who engaged in heavy binge drinking required more trials to reach habituation and showed greater variability in performance, reflecting impaired information processing and neural instability [55]. Long‐term cognitive outcomes appear to depend on cumulative exposure rather than a single critical window. In an experimental study, rats exposed to alcohol across multiple developmental stages exhibited significant impairments in spatial learning, whereas exposure limited to a single developmental period produced no lasting effects [56]. These findings suggest that repeated or sustained exposure amplifies neurodevelopmental vulnerability. At the molecular level, ethanol consistently interferes with neurotrophic signaling pathways. Chronic prenatal ethanol exposure reduced brain‐derived neurotrophic factor (BDNF) and nerve growth factor (NGF) levels and disrupted cholinergic neurons in key brain regions, leading to behavioral deficits, whereas exposure to red wine containing equivalent alcohol levels produced only minor changes [57]. Dose‐dependent reductions in BDNF expression and TrkB receptor phosphorylation have also been demonstrated in experimental models of PAE [58]. Experimental neuroprotection studies further support the central role of oxidative stress. Activity‐dependent neurotrophic factor‐12 (ADNF‐12) has been shown to attenuate ethanol‐induced developmental damage [59], and additional studies report protective effects of antioxidants such as EGCG, curcumin, resveratrol, melatonin, α‐lipoic acid, and silybin against ethanol‐induced oxidative injury and apoptosis [60, 61]. Persistent oxidative stress extending into adulthood has also been documented in the hypothalamus following prenatal ethanol exposure [62], with region‐specific damage partially reversible by omega‐3 supplementation [63]. Moreover, ethanol can be locally metabolized to acetaldehyde in the brain via a catalase‐dependent mechanism, suggesting a direct contribution of brain‐derived acetaldehyde to FASD pathology [64]. Collectively, these findings illustrate that PAE disrupts brain development through converging mechanisms involving oxidative stress, mitochondrial dysfunction, impaired neurotrophic signaling, vascular vulnerability, and altered glial‐neuronal interactions, ultimately contributing to long‐term cognitive and behavioral impairments (Table 3).

##### 3.2.2.2 Neural Tube

PAE affects early embryonic structures such as the neural tube and neural crest, leading to a range of developmental disruptions in the central nervous system and craniofacial regions.

Together, the studies summarized below indicate that ethanol primarily interferes with tightly regulated signaling and migration processes during early neurodevelopment, resulting in both neural tube defects and craniofacial abnormalities.

In one study, it was demonstrated that retinoic acid signaling plays a crucial role in neural tube closure and that its disruption can effectively induce neural tube defects (NTDs). Using *Xenopus* embryos, researchers applied retinoic acid biosynthesis inhibitors ethanol, DEAB (4‐(Diethylamino) benzaldehyde), and citral and observed the onset of NTDs. Ethanol exposure, in particular, suppressed retinoic acid signaling, leading to abnormal expression of neural regulatory genes, increased proliferation of neural progenitor cells, and expansion of the neural plate. These findings suggest that PAE may contribute to NTD formation by interfering with retinoic acid–mediated neurodevelopmental processes [41].

This study highlights disruption of morphogen signaling as an early and central mechanism underlying ethanol‐induced neural tube pathology.

In C57Bl/6J mouse embryos, ethanol exposure on gestational day 8 induced significant cell death in the anterior neural plate within 12 h. This early damage was linked to craniofacial abnormalities such as exencephaly, arhinencephaly, pituitary dysplasia, maxillary hypoplasia, and cleft lip. These findings suggest that ethanol disrupts both neural and craniofacial development by targeting critical cell populations during early embryogenesis [65].

The rapid onset of damage following early exposure underscores the presence of narrow developmental windows of heightened vulnerability.

In another study using chick embryos, a single ethanol dose was administered at various stages of cranial neural crest cell (CNCC) development. Early exposure during gastrulation or neurulation caused significant CNCC loss and severe craniofacial malformations, whereas later exposure produced milder effects. These findings emphasize the stage‐specific vulnerability of CNCCs to ethanol and may help explain the variability in craniofacial anomalies observed in FAS [70].

This stage dependency further supports the concept that timing of exposure critically shapes phenotypic severity.

Similarly, *Xenopus laevis* embryos exposed to ethanol during the neural crest migration phase exhibited craniofacial abnormalities, including microcephaly. Ethanol induced apoptosis and elevated homocysteine levels, while treatment with 5‐methyltetrahydrofolate mitigated these effects, suggesting a potential protective role in FASDs [71].

The partial rescue observed in this model indicates that ethanol‐induced neural crest injury is biologically modifiable during early development.

In a study, PAE during the neurulation period suppressed Sonic Hedgehog (Shh) signaling and disrupted primary cilia function, which is essential for Shh pathway activity. These findings support a transient ciliopathy model as a possible mechanism for alcohol‐induced craniofacial and CNS defects [66].

This mechanism provides an additional link between disrupted signaling pathways and defective neural patterning following ethanol exposure.

To investigate serotonergic system development, a study exposed female Sprague–Dawley rats to ethanol 6 weeks before mating and throughout gestation via a liquid diet. Between gestational days 13 and 20, rats received saline or ipsapirone, a 5‐HT1A receptor agonist. On the fifth day postpartum, offspring brains were analyzed via immunohistochemistry. Ethanol caused a 28%–40% loss of serotonin‐positive neurons in brainstem regions (dorsal raphe, median raphe, B9), while ipsapirone treatment prevented this loss, suggesting neurotrophic support from 5‐HT1A agonism [67].

These findings suggest that disruption of neurotransmitter‐related trophic signaling contributes to ethanol‐induced neuronal loss during development.

Complementary to these findings, another study showed that ethanol‐induced cell death in fetal rhombencephalic and serotonergic neurons was mitigated by the astrocyte‐derived protein S100B. S100B's neuroprotective effect was linked to PI3K/pAkt signaling activation and upregulation of survival genes like XIAP and Bcl‐2, while the MAPK pathway did not contribute [68].

This study further supports the role of glia‐derived survival signals in modulating ethanol‐induced neurotoxicity.

To further investigate ethanol's effects on neuronal migration, a study used in vitro and in vivo models with postnatal cerebellar granule cells labeled with the fluorescent stain DiI. Ethanol slowed cell migration, decreased intracellular Ca^2+^ and cGMP, and increased cAMP. These effects were reversible by pharmacological modulation, suggesting second messenger signaling as a key mechanism in ethanol‐induced migration defects [69].

Impaired neuronal migration represents a convergent outcome of multiple ethanol‐sensitive signaling pathways during early brain development.

Together, these findings highlight the complexity of ethanol's impact on early neural development (Table 3).

Collectively, the evidence indicates that PAE disrupts neural tube formation and neural crest development through interrelated effects on morphogen signaling, cell survival, and coordinated neuronal migration.

##### 3.2.2.3 Neural Crest

In another study using chick embryos, a single ethanol dose was administered at various stages of cranial neural crest cell (CNCC) development. Early exposure during gastrulation or neurulation caused significant CNCC loss and severe craniofacial malformations, whereas later exposure produced milder effects. These findings emphasize the stage‐specific vulnerability of CNCCs to ethanol and may help explain the variability in craniofacial anomalies observed in FAS [70].

These results indicate that the timing of ethanol exposure critically determines the extent of neural crest cell loss and subsequent craniofacial patterning defects.

Similarly, *Xenopus laevis* embryos exposed to ethanol during the neural crest migration phase exhibited craniofacial abnormalities, including microcephaly.

Given the migratory nature of neural crest cells, ethanol‐induced disruption at this stage is likely to have widespread consequences beyond craniofacial structures, affecting multiple organ systems derived from this cell population.

#### 3.2.3 Visual System

PAE has been consistently linked to a broad spectrum of structural and functional abnormalities in the visual system. These effects include both primary ocular malformations and secondary neurodevelopmental impairments that contribute to visual dysfunction. This dual involvement indicates that visual deficits in FASD reflect combined damage to ocular structures and central visual pathways rather than isolated eye abnormalities. Children with FAS commonly exhibit ocular abnormalities affecting all parts of the eye, including optic nerve hypoplasia, retinal vessel tortuosity, microphthalmia, and strabismus. Periocular facial features such as short palpebral fissures, epicanthus, and ptosis are frequently observed. Given the high prevalence of visual and ocular defects, ophthalmologic examination plays a key role in diagnosis and early intervention [104].

In a long‐term follow‐up of 25 children with FAS, nearly all showed persistent ophthalmological abnormalities, most commonly optic nerve hypoplasia and strabismus. While eye malformations remained stable, severe visual impairment continued in some cases. Despite supportive interventions, neurodevelopmental deficits persisted. These findings suggest that visual system involvement in FASD is not transient but represents a lasting consequence of early developmental injury. These results highlight the diagnostic and clinical importance of routine ophthalmologic evaluation in FAS [75].

In avian embryo models, PAE alters flow dynamics that reshape valvuloseptal morphogenesis, contributing to cardiac malformations, and suppresses Wnt and Shh signaling, thereby disrupting neural crest migration, expansion, and survival processes critical for craniofacial, retinal, and optic nerve development [72]. This observation provides a mechanistic link between neural crest dysregulation and concurrent cardiac and visual system abnormalities observed in FASD. Anterior segment anomalies such as Peter's and Axenfeld's were observed in children with FAS. The findings suggest that alcohol may exert teratogenic effects during critical periods of ocular development [73]. Together, these structural anomalies indicate heightened vulnerability of early ocular morphogenesis to ethanol exposure.

The FASD Eye Code is a complementary ophthalmological tool developed to assist in diagnosing FASD. In a clinical validation study involving 21 children with FASD and 21 matched controls, the tool demonstrated high specificity (100% at a cut‐off score ≥ 9 and 95% at ≥ 8) and moderate sensitivity (38% and 52%, respectively). While no significant differences were observed in visual perception problems between groups, the FASD Eye Code effectively identified ophthalmological abnormalities associated with FASD, supporting its role as a useful adjunct in clinical evaluation [76]. This supports the use of structured ocular assessments as part of multidisciplinary FASD diagnosis.

In a large multi‐site study, children diagnosed with FASD (*n* = 71), children with PAE but without an FASD diagnosis (*n* = 20), and typically developing controls (*n* = 111) were assessed using both standardized psychometric tests and saccadic eye movement tasks. The FASD group showed significant impairments in visuospatial processing and working memory. Poor performance in digit span, block recall, and animal sorting tasks significantly correlated with increased error rates in memory‐guided eye movement tasks. Together, these findings indicate that eye movement paradigms capture higher‐order visual cognitive dysfunction associated with PAE and may serve as sensitive tools for assessing overlapping domains of brain function [74] (Table 3).

#### 3.2.4 Cardiovascular System

Several congenital heart abnormalities associated with PAE involve neural crest‐derived structures, underscoring the role of impaired neural crest cell migration and differentiation in ethanol‐induced cardiovascular defects [78].

PAE can adversely affect the cardiovascular system by disrupting heart morphogenesis, vascular development, and hemodynamic function through multiple mechanistic pathways. Fetal alcohol exposure can lead to severe structural heart defects. Importantly, these effects appear to vary depending on developmental timing, genetic background, and the specific molecular pathways affected. In a zebrafish model chosen for its conserved cardiac development with higher vertebrates, embryos were exposed to 0.5% ethanol, resulting in marked alterations in heart morphology and volume, as well as impaired cardiac conduction characterized by delayed isoproterenol response and absence of carbachol‐induced bradycardia. These findings highlight the zebrafish as a valuable model for investigating FAS related cardiac malformations [83]. Another study demonstrated that acute ethanol exposure during gastrulation in pregnant mice disrupted Wnt/β‐catenin signaling, which resulted in myocardial wall abnormalities and valve defects. Notably, folic acid supplementation prevented these cardiac malformations and restored normal gene expression, suggesting a protective role for folate against ethanol‐induced cardiac teratogenesis [77]. This finding underscores the sensitivity of early cardiogenic signaling pathways to ethanol and highlights the potential for nutritional modulation of teratogenic risk. In a zebrafish study, prenatal ethanol exposure was found to disrupt multiple stages of heart development, with the endocardial cushion being particularly sensitive. Folic acid supplementation effectively prevented these ethanol‐induced defects in both the myocardium and endocardium, while retinoic acid offered only partial protection. The differential efficacy of these interventions further suggests that ethanol‐induced cardiac defects arise through distinct and partially overlapping molecular mechanisms. These findings emphasize the multifactorial mechanisms of ethanol‐induced cardiac defects and highlight folic acid as a potential protective agent [84]. In a study, the developmental toxicity of ethanol in chick embryos and the protective effects of vitamin C and folic acid were investigated using both in vitro and in vivo models. Ethanol exposure resulted in growth retardation, craniofacial abnormalities, and cardiac defects. However, supplementation with vitamin C (100 μM) and folic acid (1 mM) significantly mitigated these effects, likely through their antioxidant mechanisms [79]. These results support a role for oxidative stress in ethanol‐induced cardiac injury and suggest that antioxidant capacity may influence phenotypic severity. Ethanol exposure in chick embryos from different genetic backgrounds resulted in differential cardiac outcomes. While some strains were able to compensate for ethanol‐induced cardiac neural crest cell death and develop normally, B300 strain embryos exhibited ventricular thinning, chamber dilation, and reduced myosin content, indicating impaired myocardial maturation. These stage‐specific effects occurred independently of neural crest apoptosis and highlight the importance of genetic susceptibility in ethanol teratogenicity [78]. This variability emphasizes that host genetic factors can modulate both the extent and nature of cardiovascular damage following PAE. Dysplastic pulmonary arteries accompanying ventricular septal defects were reported in two infants diagnosed with FAS, as revealed by cardiac catheterization and angiography. These findings underscore both the frequency and heterogeneity of cardiovascular malformations associated with FAS and emphasize that some anomalies, such as pulmonary artery dysplasia, may be clinically silent without advanced imaging [85]. Thus, subclinical vascular abnormalities may be underrecognized in affected individuals. In a study investigating the effects of PAE during angiogenesis, ethanol was shown to impair the collateral formation of blood vessels. Between the 30th and 38th weeks of gestation, a loss of radial organization in microvascular networks was observed, indicating disrupted vascular remodeling during a critical developmental window [80]. These findings suggest that ethanol not only affects cardiac structure but also interferes with later‐stage vascular patterning. Late‐gestation alcohol exposure in sheep leads to endothelial and smooth muscle dysfunction, accompanied by increased arterial stiffness in cerebral, coronary, and renal arteries. These vascular impairments persist postnatally, potentially increasing the risk of long‐term cardiovascular disease [53]. This raises the possibility that prenatal ethanol exposure may predispose individuals to cardiovascular morbidity beyond the perinatal period. Several studies in pregnant baboons further explored the impact of PAE on fetal cardiovascular and cerebrovascular physiology. Three intra‐abdominal ethanol infusions administered during the second trimester resulted in peak maternal blood alcohol levels of 80 mg/dL. Doppler ultrasonography revealed significant reductions in fetal abdominal and head circumferences without changes in femur length. Transient decreases in systolic velocities of the anterior and middle cerebral arteries were observed during intoxication, although these changes resolved over time. These findings suggest that alcohol may exert temporally distinct effects on fetal growth and cerebral vascular function, independent of cardiac output [86]. Such transient hemodynamic alterations may nevertheless have lasting developmental consequences.

In a related study, PAE caused significant vasodilation in fetal cerebral arteries via activation of endocannabinoid receptors, particularly CB2. Although no changes in receptor expression were observed, CB2‐mediated signaling was enhanced, altering vascular tone [81]. This indicates that ethanol can modulate vascular function through receptor‐level signaling rather than changes in receptor abundance. Mid‐gestation alcohol exposure in baboons induced transient dilation of fetal cerebral arteries through cannabinoid receptor pathways. However, this response was absent in the near term. Furthermore, while both maternal and fetal cerebral arteries showed similar tone and reactivity, they failed to exhibit ethanol‐induced dilation at later gestational stages. These results imply that alcohol's effects on the fetal cerebrovasculature via the endocannabinoid system are temporally restricted, diminishing with advancing gestation, which may partially explain inconsistencies across studies examining vascular outcomes [82]. Taken together, the findings across experimental and clinical studies indicate that the cardiovascular effects of PAE are highly context dependent rather than inherently contradictory. Differences in species, genetic background, developmental timing, and the specific molecular pathways targeted by ethanol critically shape the resulting phenotype. Early disruptions affecting cardiogenic signaling and neural crest‐derived structures tend to produce permanent structural malformations, whereas alcohol exposure during later gestational windows more frequently alters vascular tone, endothelial function, and hemodynamic regulation, some of which may be transient or clinically silent. This variability highlights that ethanol‐induced cardiovascular abnormalities reflect a spectrum of outcomes governed by developmental stage and biological susceptibility, underscoring the need to interpret these effects within precise temporal and mechanistic frameworks (Table 3).

#### 3.2.5 Liver

PAE can adversely affect fetal liver development and metabolic function through multiple mechanisms, including disruption of glycogen metabolism, suppression of hepatocyte proliferation, oxidative stress, altered enzyme activity, fibrosis, and hormonal signaling disturbances. In a study conducted on rats during term pregnancy, chronic ethanol intake combined with short‐term fasting before birth was associated with fetal growth retardation, hypoglycemia, and a marked reduction in hepatic glycogen stores. Moreover, significant decreases were observed in both active and total levels of glycogen synthase and phosphorylase enzymes critical to glycogen metabolism. Collectively, these alterations indicate that prenatal ethanol exposure compromises neonatal energy balance, with potential long‐term consequences for glucose homeostasis and hepatic reserve capacity [87].

To investigate the developmental impact of PAE on the liver, pregnant rats were fed a liquid diet containing 36% ethanol throughout gestation. Offspring were examined on postnatal days 1, 3, 7, and 14. Histological assessments using hematoxylin–eosin staining revealed no overt structural damage. However, a significant decrease in liver‐to‐body weight ratio and DNA synthesis rate (measured via [^3^H] thymidine incorporation) was observed, despite unchanged total liver protein content, normal ornithine decarboxylase (ODC) activity, and unaltered expression of GHr, IGF‐I, IGF‐II, and IGFBP1‐4 mRNAs [88]. This pattern suggests that PAE restricts hepatic growth primarily through impaired cellular proliferation rather than through disruption of polyamine synthesis or growth factor signaling.

In another experimental study examining prenatal ethanol exposure, the activities of various biochemical enzymes were evaluated in the liver and brain tissues of rat pups born to alcohol‐exposed mothers. In the liver, increased activity of enolase and ADH was observed, whereas other enzyme activities were reduced. Conversely, in the brain, reductions were noted in SOD, enolase isoenzymes, glutamine synthetase, alcohol and ADH, and ATPase activities. Notably, these impairments persisted even after maternal alcohol consumption was discontinued during pregnancy or lactation. Such persistence points to sustained metabolic and oxidative vulnerability induced by early ethanol exposure, underscoring the toxic effects of alcohol and acetaldehyde and highlighting the role of diminished antioxidant defense particularly reduced SOD activity in the pathogenesis of FAS [89].

Histopathological alterations consistent with alcoholic liver disease have also been documented in children with FAS. In one case, light and electron microscopic analysis of a liver biopsy from a 17‐month‐old child with FAS revealed parenchymal steatosis along with portal and perisinusoidal fibrosis. Ultrastructural examination showed medium to large collagen fibers, myofibroblasts, occasional Ito cells, and subendothelial basement membrane‐like material within perisinusoidal spaces [90]. These features mirror fibrotic changes observed in adult and primate alcoholic liver disease, supporting a shared pathogenic continuum extending from prenatal exposure into postnatal life.

Recent studies have explored the protective potential of dietary interventions. PAE in pregnant rats impairs placental development and disrupts insulin/IGF‐1 signaling pathways, contributing to fetal growth restriction and features characteristic of FASD. Substitution of dietary casein with soy protein isolate mitigated many adverse outcomes, including fetal loss, placental dysfunction, and Akt/PRAS40 pathway signaling deficits [92]. Similarly, chronic PAE suppresses the expression of insulin, IGF‐1, IGF‐1R, IRS1/2, ASPH, Notch, and Hes1 genes in placental tissue, together with reductions in ASPH protein levels. Replacing casein with soy protein largely reversed these impairments by upregulating Igf2, Irs4, Notch, and Hes1 expression [91]. These findings support a modulatory role for maternal nutrition in shaping the severity of ethanol‐induced hepatic and placental dysfunction.

Disruption of iron metabolism represents another pathway through which PAE affects fetal liver and systemic development. In nulliparous Long‐Evans rats, PAE increased iron accumulation in the fetal liver irrespective of maternal iron status, while concurrently reducing brain iron levels as well as ferritin, transferrin, and transferrin receptor concentrations [93]. Such redistribution of iron links hepatic dysfunction to secondary neurodevelopmental vulnerability, particularly under conditions of maternal iron deficiency.

Early alcohol exposure (EAE) was also shown to impair liver and adipose tissue development in a zebrafish model using a multimodal approach including RNA‐Seq, in situ hybridization, confocal microscopy, hematoxylin‐eosin staining, lipid‐specific dyes (Nile red and Oil Red O), qRT‐PCR, behavioral testing, swim chamber performance, and morphometric analyses. EAE resulted in reduced liver and bile duct size, diminished hepatocyte number, and impaired differentiation [94]. Taken together, the available evidence indicates that PAE predisposes offspring to long‐term metabolic dysregulation through combined structural, enzymatic, and signaling‐level disturbances of hepatic development (Table 3).

#### 3.2.6 Lung

PAE has been associated with multiple adverse effects on fetal and neonatal lung development. These include oxidative stress, impaired surfactant production, abnormal collagen deposition, altered phospholipid composition, lung growth restriction, and increased susceptibility to respiratory disease. Together, these alterations indicate that PAE affects both the structural maturation and functional readiness of the developing lung. Experimental and clinical findings suggest that some of these effects may be transient, while others may result in long‐term pulmonary consequences. In a rat model, prenatal ethanol exposure significantly impaired fetal lung development. Ethanol‐exposed fetuses exhibited reduced lung weight and decreased DNA, RNA, and protein content. Histological analysis revealed lung hypoplasia with fewer and smaller cells, indicating a disproportionate restriction of lung growth relative to overall fetal development [97]. This finding suggests that ethanol interferes with fundamental cellular proliferation processes during critical windows of pulmonary development, potentially limiting postnatal respiratory reserve. Further supporting these observations, PAE during late gestation in sheep increased collagen deposition, reduced surfactant protein A and B expression, and altered phospholipid composition in fetal lungs. These changes were linked to decreased lung compliance and impaired innate immune responses at birth [53]. The combined disruption of extracellular matrix composition and surfactant homeostasis highlights a dual structural‐functional vulnerability of the ethanol‐exposed lung. Although some alterations appeared reversible postnatally, the transient disruption of pulmonary function may predispose neonates to respiratory complications.

To assess the persistence of ethanol‐induced alterations in lung development, a follow‐up study in a sheep model found that prenatal ethanol exposure increased collagen deposition and disrupted surfactant protein expression in fetal lungs. At 9 weeks postnatally, most structural, inflammatory, and surfactant parameters normalized; however, surfactant protein D mRNA expression remained elevated, suggesting lingering changes in pulmonary innate immunity [96]. This selective persistence of immune‐related molecular alterations suggests that prenatal ethanol exposure may reprogram aspects of pulmonary host defense beyond the neonatal period. Oxidative stress has been identified as a key mechanism contributing to alcohol‐induced lung injury. The combined and separate effects of ethanol and benzo[a]pyrene (B[a]P) on lung and brain tissues were evaluated in male Sprague–Dawley rats. Oxidative stress was evidenced by decreased SOD activity, reduced GSH levels, and increased malondialdehyde (MDA) concentrations. Histological analysis showed marked damage in lung tissue, more severe than in the brain [95]. These results indicate that the lung may be particularly susceptible to oxidative injury induced by ethanol, especially in the presence of additional environmental toxicants. Although most studies highlight the detrimental impact of alcohol on fetal lung development, a decreased incidence of respiratory distress syndrome (RDS) in preterm infants (< 37 weeks' gestation) born to mothers who consumed alcohol during pregnancy was reported. The authors hypothesized that alcohol might accelerate fetal lung maturation. This association remained statistically significant (*p* < 0.02) even after adjusting for confounding variables such as gestational age, birth weight, and maternal smoking [98]. This apparently protective association contrasts with experimental evidence of lung injury and may reflect confounding clinical factors or transient acceleration of surfactant production that does not equate to healthy lung development. The biological plausibility and mechanisms behind this observation remain unclear and warrant further investigation. A 6‐month‐old male infant who was found dead at home was described in a clinical case report. The mother had reportedly consumed alcohol throughout pregnancy. The infant exhibited signs of FAS at autopsy, including postnatal growth retardation, multiple facial anomalies, and abnormal brain structures. The cause of death was identified as severe aspiration pneumonia [99]. This case underscores the potential long‐term vulnerability of infants with FAS to severe respiratory complications, possibly reflecting compromised pulmonary and immune function. Collectively, the available evidence suggests that PAE disrupts lung development through converging mechanisms involving impaired growth, extracellular matrix remodeling, oxidative stress, and altered innate immunity, with both transient and persistent consequences depending on exposure timing and severity (Table 3).

#### 3.2.7 Kidney

PAE has been associated with structural and developmental alterations in the fetal renal system, which may contribute to long‐term health risks despite limited immediate functional consequences. This pattern suggests that renal involvement in PAE may remain clinically silent in early life while conferring latent vulnerability. A high incidence of hydronephrosis was observed in the offspring of mice exposed to ethanol during gestation. This finding suggests that prenatal ethanol exposure may impair normal renal morphogenesis, potentially resulting in structural abnormalities of the urinary tract [100]. Such abnormalities point to disrupted ureteric bud branching or nephrogenic patterning during critical windows of kidney development. Similarly, in a sheep model, prolonged maternal alcohol consumption during late gestation led to an 11% reduction in total nephron number in the fetus, despite the absence of overt renal injury or functional impairment at that stage. Although immediate physiological consequences appeared negligible, this nephron deficit may predispose individuals to hypertension and other cardiovascular complications later in life, particularly when compounded by environmental or metabolic stressors such as poor nutrition or obesity [53]. This observation aligns with the developmental origins of health and disease (DOHaD) hypothesis, linking reduced nephron endowment to long‐term cardiovascular risk. Infants with FAS exhibited impaired distal tubular acidification and reduced potassium excretion, despite otherwise normal renal function. Compared to controls, FAS patients showed significantly higher minimum urine pH and lower net acid and potassium excretion following acid and bicarbonate loading. These abnormalities were not related to aldosterone deficiency and were partially corrected with chlorothiazide treatment, suggesting a defect in distal tubular function [101]. These findings indicate a selective tubular vulnerability rather than global renal failure in ethanol‐exposed infants. Supporting these findings, similar renal abnormalities were reported in older FAS patients, including impaired maximal urinary concentration after water deprivation and vasopressin administration, as well as defective urinary acidification following ammonium chloride loading. Together, these studies highlight a consistent pattern of subclinical renal tubular dysfunction associated with PAE [102]. The persistence of these abnormalities across age groups suggests that prenatal ethanol exposure may induce long‐lasting alterations in tubular maturation or regulatory signaling pathways. These findings underscore the importance of further investigation into the long‐term renal consequences of PAE (Table 3). In particular, longitudinal studies are needed to clarify how early structural and tubular alterations translate into adult renal and cardiovascular disease risk.

#### 3.2.8 Limb

PAE has been associated with a spectrum of limb abnormalities, often co‐occurring with defects in other organ systems, suggesting a systemic teratogenic effect during early embryogenesis. This co‐occurrence supports the concept that limb anomalies in FAS are rarely isolated findings but rather reflect broader disruptions of developmental patterning during critical windows of organogenesis. In a study, pregnant mice were fed ethanol‐containing liquid diets during early gestation, while control groups received either standard laboratory diets or isocaloric diets in which sucrose replaced ethanol. Upon examination at term, a clear dose‐related increase in congenital malformations was observed. These anomalies involved the skeletal, neurological, urogenital, and cardiovascular systems. Limb defects such as adactyly, ectrodactyly, and syndactyly, along with ocular malformations such as anophthalmia and microphthalmia, were commonly observed in both alcohol‐exposed and control groups [103]. The simultaneous involvement of limb and ocular structures further suggests that ethanol interferes with shared developmental pathways, including mesenchymal proliferation and patterning signals active during early gestation. Further exploration of these patterns may contribute to a more nuanced understanding of alcohol‐related teratogenic outcomes (Table 3). From a narrative perspective, limb abnormalities may therefore serve as visible markers of widespread embryonic vulnerability rather than isolated skeletal defects.

### 3.3 Other Impacts of PAE

#### 3.3.1 Endocrine and Metabolic Effects

Beyond its structural teratogenic effects, PAE has also been associated with alterations in the fetal endocrine system and metabolic regulation, altering hormone sensitivity, stress responses, and gene networks involved in cellular energy balance. This suggests that ethanol exposure may influence not only organ morphogenesis but also systemic regulatory pathways that coordinate growth and homeostasis. A study investigated the molecular mechanisms underlying FASD caused by alcohol exposure during pregnancy. Two different mouse strains (B6J and B6N) were used, and FASD symptoms were examined following ethanol administration on the eighth day of pregnancy. B6J embryos were found to be more sensitive to alcohol. A compound called PK11195, administered alongside alcohol, reduced fetal damage in B6J embryos but slightly increased it in B6N embryos. Gene expression analyses revealed that alcohol affects gene pathways related to cellular skeleton organization, adhesion, energy metabolism, and apoptosis [105]. These strain‐dependent effects underscore the role of genetic background in modulating endocrine and metabolic vulnerability to PAE. In this study examining physiological and hormonal disorders associated with prenatal ethanol exposure, it was shown that “handling” intervention applied in the early stages alleviated growth retardation, thermoregulation disorders, and increased stress responses caused by ethanol. In particular, hypothermia was reduced in male rats, while the corticosterone response was balanced in females [106]. These findings suggest that early‐life environmental modulation can partially normalize dysregulated hypothalamus–pituitary–adrenal (HPA) axis activity induced by PAE, highlighting the plasticity of endocrine systems during development. These insights reflect the complexity of endocrine and metabolic regulation under the influence of PAE (Table 3). Taken together, the available findings suggest that endocrine and metabolic disturbances may contribute to the broader features of FASD, linking molecular alterations to long‐term physiological outcomes.

#### 3.3.2 Epigenetic and Long‐Term Effects

Although the primary focus of this review is on organ‐specific histopathological alterations induced by PAE, increasing evidence indicates that such early structural and cellular injuries may exert long‐lasting effects through epigenetic reprogramming. In this context epigenetic mechanisms are increasingly considered a potential link between early tissue level damage and persistent functional, cognitive, and metabolic outcomes observed later in life.

The long term effects of PAE extend beyond overt physical malformations, encompassing epigenetic modifications that may shape future behavior, cognition, and metabolism. Recent findings suggest that although light‐to‐moderate levels of alcohol consumption do not lead to FAS, they may still contribute to epigenetic changes that predispose individuals to adverse health outcomes such as metabolic disorders and an increased propensity to consume alcohol in adolescence. PAE has been reported to be associated with epigenetic alterations affecting genes that regulate lipid and glucose metabolism, leading to persistent metabolic abnormalities. Moreover, PAE may program offspring for increased alcohol intake later in life by enhancing alcohol palatability and acceptance through associative learning mechanisms, thereby acting as a potential “second hit” in the development of alcohol‐induced liver disease [38].

These epigenetic changes are thought to originate from alcohol‐induced disruptions in cellular differentiation, oxidative stress responses, and tissue architecture during critical periods of organogenesis, thereby providing a mechanistic bridge between early histopathological injury and long‐term functional vulnerability. In one study, 12 children diagnosed with FASDs were examined to assess long‐term neurocognitive outcomes. Nonverbal intelligence was evaluated using the Leiter‐3 test, while executive functions were assessed with the Behavior Rating Inventory of Executive Function (BRIEF) and temperament questionnaires completed by parents. The children also underwent a continuous performance test to evaluate attention and inhibitory control. Notably, performance differences between the first and second halves of the task were significantly associated with parent‐reported executive dysfunction, suggesting that temporal changes in task performance may more accurately reflect impairments in daily functioning [107]. Such findings are increasingly interpreted as functional manifestations of earlier structural and epigenetic alterations in the developing brain rather than isolated cognitive phenomena. In another study, 40 individuals diagnosed with FASD were evaluated for sleep‐related disturbances, which are increasingly recognized as a downstream consequence of altered neurodevelopment. Initial screening using the Child Sleep Habits Questionnaire identified individuals at high risk, who subsequently underwent polysomnography (PSG). Comprehensive PSG assessment, including electroencephalography, electrooculography, electromyography, electrocardiography, respiratory monitoring, and pulse oximetry, revealed disrupted sleep architecture and a high prevalence of apnea and hypopnea events. These findings support clinical observations that sleep disorders represent a significant and persistent health issue in individuals with FASD [108]. Such disturbances may reflect long‐term functional consequences of prenatal alcohol‐induced structural and molecular alterations within central regulatory circuits governing sleep and circadian rhythm. Collectively, these studies suggest that epigenetic dysregulation may represent one of the mechanisms through which early histopathological damage induced by PAE translates into sustained neurobehavioral, metabolic, and physiological vulnerabilities across the lifespan. These observations support the consideration of epigenetic perspectives when interpreting organ‐specific histopathological findings associated with PAE (Table 4).

**TABLE 4:** Other impacts of prenatal alcohol exposure (PAE).

Category	Mechanisms of action	Observed effects
Endocrine and metabolic effects	− Ethanol alters gene expression pathways related to cytoskeleton organization, cell adhesion, energy metabolism, and apoptosis, contributing to structural and functional developmental abnormalities [105]. − Early life “handling” intervention mitigates physiological and hormonal disturbances induced by prenatal ethanol exposure, improving thermoregulation and modulating stress hormone responses [106]	− Increased sensitivity to ethanol‐induced teratogenesis in B6J embryos compared to B6N; PK11195 administration reduced fetal damage in B6J but slightly increased it in B6N [105]. − Handling reduced hypothermia in male offspring and normalized corticosterone responses in female offspring; also alleviated growth retardation and stress‐related abnormalities [106]
Epigenetic and long‐term effects	− Epigenetic modifications affecting genes that regulate lipid and glucose metabolism, leading to metabolic dysregulation [38]. − Neurodevelopmental programming that increases the palatability and acceptance of alcohol, enhancing the likelihood of heavier alcohol consumption later in life [38]	− Increased risk of metabolic disorders and heightened susceptibility to alcohol‐induced liver disease [38]. − Executive function impairments in children with fetal alcohol syndrome disease (FASD), with task performance changes over time linked to daily living skill deficits [107]. − High prevalence of sleep disturbances in individuals with fetal alcohol syndrome disease (FASD), including disrupted sleep architecture and apnea/hypopnea events [108]

*Note:* This table summarizes the endocrine/metabolic and epigenetic or long‐term outcomes associated with PAE that were identified during the primary histopathology‐focused literature screening. These findings are presented as secondary and supportive consequences rather than primary organ‐specific histopathological endpoints and complement the organ‐based results shown in Table 3.

## 4 Discussion

This narrative review integrates experimental and clinical evidence accumulated over more than five decades to delineate the organ‐specific histopathological consequences of PAE. Collectively, the findings confirm that ethanol is a potent teratogen capable of inducing a wide spectrum of structural, cellular, and long‐term developmental abnormalities. Importantly, this synthesis highlights both distinct patterns of injury affecting individual organs and shared, nonorgan‐specific pathogenic mechanisms that link these abnormalities across multiple fetal systems.

Placental pathology emerged as one of the earliest and most consistent manifestations of PAE. Experimental studies have documented villous immaturity, trophoblastic hypertrophy, vascular congestion, fibrosis, and inflammatory alterations in a dose‐ and timing‐dependent manner [44, 45]. These findings are reinforced by recent human data demonstrating placental inflammation and altered cellular composition associated with maternal alcohol consumption [43]. Such placental abnormalities are likely to compromise maternofetal nutrient and oxygen exchange, thereby contributing to intrauterine growth restriction and increasing susceptibility to downstream organ‐specific injury.

Among fetal organs, the CNS was the most consistently and severely affected. Across experimental models, PAE was associated with neuronal apoptosis, glial activation, disrupted white matter architecture, neurotransmitter imbalance, and impaired neurodevelopmental signaling. Mechanistic studies implicate oxidative stress, mitochondrial dysfunction, altered BDNF–TrkB signaling, and disruption of retinoic acid and Sonic hedgehog (Shh) pathways as central contributors to CNS vulnerability [32, 41, 66]. Importantly, the neural tube and neural crest represent distinct developmental tissues that are differentially affected by ethanol exposure. Neural crest cells appear particularly sensitive to ethanol‐induced apoptosis and migration defects. Because neural crest derivatives contribute not only to craniofacial structures but also to components of the cardiac outflow tract, their disruption provides a unifying developmental explanation for the frequent co‐occurrence of craniofacial anomalies and congenital heart defects observed following PAE. Experimental studies have demonstrated that ethanol exposure selectively increases apoptosis in neural crest populations and disrupts their migratory capacity, thereby affecting multiple organ systems derived from this lineage, including craniofacial structures and the cardiac outflow tract [66, 78].

Cardiovascular findings in the reviewed literature included structural malformations, altered vascular development, and impaired tissue organization. Some of these abnormalities, particularly those involving the cardiac outflow tract, are likely linked to ethanol‐induced disturbances in cardiac neural crest migration, further reinforcing the interconnected nature of neurodevelopmental and cardiac pathology [78]. Beyond the CNS and heart, several additional organs exhibited characteristic histopathological patterns. The liver demonstrated glycogen depletion, steatosis, and early fibrotic changes; the lung showed growth restriction and impaired alveolar development; the kidney exhibited tubular alterations and reduced nephron endowment; and, although less frequently reported, ocular abnormalities and limb defects were also documented [33, 35, 39, 41]. While each organ displayed unique pathological features, the recurrent involvement of oxidative injury, inflammation, and disrupted developmental signaling across systems indicates that PAE exerts both localized and systemic teratogenic effects.

The severity and spectrum of these abnormalities were modulated by multiple interacting factors, including the dose, pattern, and timing of alcohol exposure, as well as maternal nutrition, socioeconomic conditions, and genetic susceptibility. Although some epidemiological studies report no clear association between low‐to‐moderate alcohol consumption and major structural malformations [15], experimental evidence consistently demonstrates that binge or chronic exposure induces pronounced multi‐organ pathology. Moreover, the presence of shared pathogenic mechanisms such as oxidative stress, endocrine dysregulation, and altered developmental signaling suggests that even in the absence of overt malformations, subtle cellular and functional impairments may persist into postnatal life [32, 33]. In addition to acute structural injury, growing evidence indicates that PAE induces persistent epigenetic modifications that may alter gene expression long after birth. These changes have been linked to long‐term neurobehavioral, metabolic, and cardiovascular vulnerability, suggesting that early histopathological damage may initiate lifelong functional consequences beyond the prenatal period [38, 66].

Overall, this review underscores that PAE results in a complex interplay between organ‐specific histopathological lesions and systemic developmental disruption. Together, these findings emphasize that fetal vulnerability arises not from isolated defects, but from coordinated disturbances across interconnected developmental systems. Accordingly, no safe threshold for alcohol consumption during pregnancy can be established, and complete abstinence remains the only evidence‐based preventive recommendation [5, 29].

### 4.1 Strengths and Limitations

A major strength of this review is its broad and integrative scope, combining mechanistic insights from experimental models with histopathological and clinical observations from human studies, thereby providing a comprehensive overview of the systemic impact of PAE. However, as a narrative review, no formal systematic search or selection protocol was applied, and the included studies were heterogeneous in design, exposure assessment, and outcome definitions. In addition, for certain organ systems, the number of available studies was limited, and only English‐language publications were included, which may have constrained the comprehensiveness of the review.

Future research should prioritize prospective cohort studies with precise exposure quantification, standardized histopathological endpoints, and integration of multi‐omics approaches. Moreover, interventional studies evaluating nutritional or pharmacological strategies to mitigate ethanol‐induced injury remain an important area for further investigation.

## 5 Conclusion

FAS is a preventable yet severe multisystem disorder caused by maternal alcohol use during pregnancy, affecting the brain, placenta, and multiple other organs through mechanisms such as oxidative stress, mitochondrial dysfunction, and impaired signaling. The fetus's limited metabolic capacity prolongs ethanol exposure, amplifying damage and leading to structural, functional, and behavioral deficits. Despite extensive research, FAS and other FASD remain underdiagnosed. With no safe threshold for alcohol intake, complete maternal abstinence is the most effective preventive measure, while future research should focus on elucidating molecular mechanisms and developing targeted interventions to mitigate harm.

## Ethics Statement

The authors have nothing to report.

## Consent

The authors have nothing to report.

## Conflicts of Interest

The authors declare no conflicts of interest.

## Data Availability

The data that support the findings of this study are available from the corresponding author upon reasonable request.
